# Lower-Limb Phase Angle and Muscle Mass Ratio Are Associated with Slow Timed Up and Go Performance in Community-Dwelling Japanese Adults

**DOI:** 10.3390/jcm15114388

**Published:** 2026-06-05

**Authors:** Daisuke Homma, Norio Imai, Dai Miyasaka, Moeko Yamato, Masafumi Ishisaki, Tsubasa Sugahara, Mie Yamada, Hayato Suzuki, Yoji Horigome, Atsushi Sakagami, Yoichiro Dohmae, Naoto Endo, Izumi Minato, Hiroyuki Kawashima

**Affiliations:** 1 Division of Orthopaedic Surgery, Graduate School of Medical and Dental Sciences, Niigata University, 757 Asahimachi-dori, Ichiban-cho, Chuo-ku, Niigata 951-8510, Japan; 2 Department of Rehabilitation, Niigata Bandai Hospital, 2-2-8 Yachiyo, Chuo-ku, Niigata 950-0909, Japan; 3 Division of Comprehensive Musculoskeletal Medicine, Graduate School of Medical and Dental Sciences, Niigata University, 757 Asahimachi-dori, Ichiban-cho, Chuo-ku, Niigata 951-8510, Japan; imainorio2001@yahoo.co.jp (N.I.);; 4 Division of Orthopaedic Surgery, Niigata Bandai Hospital, 2-2-8 Yachiyo, Chuo-ku, Niigata 950-0909, Japan; 5 Division of Orthopaedic Surgery, Tachikawa General Hospital, 24-1 Asahioka, Nagaoka 940-8621, Japan; 6 Department of Orthopedic Surgery, Saiseikai Niigata Kenoh Hospital, 5001-1 Kamisugoro, Sanjo 955-0091, Japan; 7 Division of Orthopaedic Surgery, Niigata Rinko Hospital, 1-114-3 Momoyamacho, Higashi-ku, Niigata 950-0051, Japan

**Keywords:** phase angle, muscle mass, bioelectrical impedance analysis, Timed Up and Go test, receiver operating characteristic

## Abstract

**Background**/**Objectives**: Lower-limb muscle quantity and quality may be associated with mobility performance, but the relationship between bioelectrical impedance analysis (BIA)-derived lower-limb indices and slow Timed Up and Go (TUG) performance remains insufficiently established. This cross-sectional study examined the associations of lower-limb muscle mass and phase angle (PhA) with TUG performance and explored ROC-derived values for slow TUG performance in 280 community-dwelling Japanese adults. **Methods**: Lower-limb muscle mass and segmental lower-limb PhA were measured using multifrequency segmental BIA and averaged across both legs. Multiple linear regression analyses were used to examine associations between TUG time and BIA-derived indices after adjusting for age and body size. Receiver operating characteristic analyses were performed for slow TUG performance, defined as TUG ≥ 9 s and ≥10.2 s, and Youden index-derived values were calculated as exploratory estimates. **Results**: Lower-limb PhA was independently associated with TUG performance across both body-size adjustment models, whereas lower-limb muscle mass was retained only in the model adjusted for BMI. For slow TUG performance, the lower-limb muscle mass-to-body weight ratio showed moderate discriminatory ability, with AUCs of 0.75 for TUG ≥ 9 s and 0.78 for TUG ≥ 10.2 s. Standardized lower-limb PhA showed AUCs of 0.65 and 0.64, respectively. The Youden index-derived standardized PhA value was Z = −1.04 for both TUG thresholds; however, these ROC-derived values were not internally or externally validated. **Conclusions**: Lower-limb BIA-derived indices, particularly segmental lower-limb PhA, were associated with TUG performance in community-dwelling Japanese adults. The ROC-derived values should be interpreted strictly as exploratory, hypothesis-generating estimates and should not be used for clinical screening or individual-level clinical decision-making without validation in larger independent cohorts.

## 1. Introduction

Population aging represents a major public health concern globally. The number of adults aged ≥ 60 years is projected to reach approximately 2.10 billion by 2050 [1]. With population aging, geriatric syndromes, such as frailty and sarcopenia, become increasingly common, underscoring the necessity of extending healthy life expectancy and maximizing the period during which individuals can live independently [2]. Age-stratified evidence indicates a marked age gradient in frailty prevalence. For instance, a systematic review and meta-analysis conducted in Japan showed that the prevalence of frailty increased progressively across older age bands, with the highest prevalence in the oldest age group (e.g., ≥85 years) [3]. Japan is a relevant setting to examine these issues because it is already experiencing advanced population aging; in 2021, adults aged ≥ 65 years accounted for 28.90% of Japan’s population [4]. Moreover, international comparisons and policy reports have indicated that the proportion of adults aged ≥ 65 years in Japan is higher than that in several high-income countries, making Japan a “front runner” and an early test case for strategies addressing rapid aging and its functional consequences [5].

Mobility is fundamental to independent living and is closely associated with healthy life expectancy. The Timed Up and Go (TUG) test is a brief, widely used measure of functional mobility [6], and slower TUG performance predicts incident disability and other adverse outcomes in community-based cohorts [7,8]. Among several performance-based tests used to evaluate mobility and fall risk, the TUG test is considered the standard practical screening test for older adults in clinical guidelines [9]. Reported TUG thresholds vary across populations. For example, Makizako et al. [8] identified TUG ≥ 9 s as a predictive cutoff value for incident disability in older Japanese adults; whereas Choo et al. [7] reported that a cutoff value of 10.20 s distinguished high physiological fall risk and a threshold value of ≥9.45 s was associated with increased risks of prevalent disability, functional decline, and incident disability in a Singaporean cohort. Collectively, these findings support the use of TUG-based thresholds as practical indicators for identifying individuals at an increased risk of mobility-related adverse events.

Objective muscle function assessments with magnetic resonance imaging [10], computed tomography [11], or electromyography [12] can provide detailed information on muscle morphology, muscle composition, and neuromuscular activity, which are relevant to TUG performance. However, these modalities are not always suitable for routine screening or community-based assessment because they require specialized equipment, trained operators, higher cost, and/or longer measurement time. In addition, some procedures may impose a greater physical burden on participants than do simple field-based assessments [13]. In contrast, bioelectrical impedance analysis (BIA) enables non-invasive and time-efficient assessment of muscle-related indicators and is feasible for routine or large-scale use [14]. International consensus guidelines for sarcopenia recognize BIA as a practical method for estimating appendicular muscle mass in clinical and population-based settings [15]. As absolute muscle mass is strongly influenced by body size, expressing muscle mass relative to body size has been adopted in major frameworks to facilitate interpretation across individuals [16]. However, clinically interpretable BIA-derived indicators related to lower-limb muscle quantity and quality, as well as their correspondence to clinically meaningful slow-TUG thresholds, remain insufficiently established, particularly in community-dwelling Japanese adults.

Muscle quality, in addition to muscle quantity, is important for mobility in aging. Although muscle mass loss is a key feature of age-related decline, longitudinal cohort studies have demonstrated that reductions in muscle strength and physical function are disproportionate to concurrent lean mass loss, indicating that factors beyond quantity contribute to functional deterioration [17,18]. Age-related changes encompass qualitative alterations, such as adipose tissue infiltration, increased fibrous tissues, and shifts in extracellular fluid distribution within skeletal muscles [14,17,18]. Accordingly, biomarkers of muscle quality may complement conventional assessments based solely on muscle quantity. The phase angle (PhA) derived from BIA is a non-invasive marker that reflects changes in tissue electrical properties and fluid distribution. Previous studies have proposed PhA as a surrogate indicator of muscle quality [13,19] and reported an association between lower PhA and frailty in community-dwelling older adults (typically ≥65 years) [20]. Furthermore, a meta-analysis involving older populations examined PhA as a screening marker for sarcopenia; however, age thresholds varied across studies [21]. Previous cutoff studies have primarily focused on sarcopenia screening or walking independence in specific populations rather than standardized mobility outcomes in adults [22,23]. Most reports have also focused on whole-body PhA, whereas only a few studies have investigated whether segmental lower-limb PhA provides task-relevant information for mobility-specific outcomes [24]. Given that PhA varies systematically by age and sex, age- and sex-referenced standardization (e.g., *Z*-scores) may support interpretation across heterogeneous populations [25,26].

These gaps are particularly relevant from a preventive perspective because muscle-related characteristics across adulthood may help identify early functional vulnerability before overt mobility limitation develops. BIA provides non-invasive and scalable indices of muscle quantity and quality, including lower-limb muscle mass and segmental PhA, and may complement, rather than replace, performance-based mobility assessments such as the TUG test. However, it remains unclear whether lower-limb BIA-derived indices are independently associated with TUG performance after accounting for age and body size, and whether exploratory ROC-derived values for these indices correspond to established slow-TUG criteria in community-dwelling Japanese adults. Therefore, this study aimed to investigate the associations of lower-limb muscle mass and segmental lower-limb PhA with TUG performance and to explore ROC-derived values for slow TUG performance. We hypothesized that lower-limb muscle mass and PhA would be independently associated with TUG performance and would show measurable, but exploratory, discriminatory ability for slow TUG performance.

## 2. Participants and Methods

### 2.1. Study Design and Measures

This cross-sectional study included 280 community-dwelling Japanese adults. Participants’ basic characteristics (age, height, and weight), BIA-derived indices, and TUG performance were measured. Body composition was assessed using BIA, from which lower-limb muscle mass (kg) and segmental lower-limb PhA (degrees) were derived as indices of muscle quantity and quality, respectively. The primary outcome was functional mobility, assessed by TUG time (s).

### 2.2. Participants

A convenience sampling approach was employed to recruit community-dwelling residents living near Niigata or Shibata City (Niigata Prefecture, Japan) between November 2019 and October 2024. These cities were selected because they constitute the primary catchment communities for our collaborating sites, enabling standardized assessments by the same trained staff throughout the study period. The eligibility criteria were as follows: (i) independent ambulation without an assistive device, (ii) independence in performing activities of daily living, (iii) no history of conditions that could substantially affect daily life (e.g., paralysis), (iv) no prominent neurological symptoms (e.g., clinically relevant numbness), (v) no implanted pacemaker, and (vi) Japanese ethnicity. In total, 292 individuals were assessed during the study period; 11 individuals aged < 20 years and 1 individual with a data entry error were excluded from the analysis, leaving 280 participants (43 men and 237 women).

Regarding sample size considerations, volunteer convenience sampling was employed in this study, and all eligible participants assessed during the recruitment period were included to maximize statistical precision. For the multiple linear regression analyses, the planned models included up to six candidate explanatory variables. In the commonly cited rule-of-thumb proposed by Green, m represents the number of explanatory variables included in the regression model [27]. In the present study, m was set to 6 because Model 2 included the largest number of candidate variables: age, sex, height, weight, lower-limb muscle mass, and lower-limb PhA. Based on this rule, the recommended minimum sample size was N ≥ 104 + m for testing individual predictors and N ≥ 50 + 8 m for evaluating overall model fit [27]. With m = 6, the recommended minimum sample size ranged from 98 to 110. The final sample size of 280 participants exceeded these minimum recommendations. As this was not an a priori sample-size-targeted study but a convenience-sampling study, we included all eligible participants assessed during the recruitment period to improve statistical precision and the stability of the regression and ROC estimates.

For ROC analysis and derivation of exploratory values using the Youden index, diagnostic performance was evaluated by reporting AUC with 95% CIs and by estimating the AUC standard error using established methods [28]. Sample size considerations for diagnostic accuracy and ROC studies have been discussed previously, emphasizing precision-based evaluation rather than fixed predictor counts [29].

### 2.3. BIA

Trained staff performed all assessments, with measurements conducted under temperature-controlled indoor conditions at local community facilities or the Niigata Bandai Hospital. PhA and muscle mass were assessed using a multifrequency, eight-electrode body composition analyzer (MC-780A-N; Tanita, Tokyo, Japan), which has been used in prior studies with comparable populations [30,31]. Before measurements, the participants removed any metal accessories (e.g., watches and jewelry), and the skin and electrodes were cleaned with alcohol to ensure stable electrode contact. The participants stood barefoot on the footplate electrodes and held the hand grips with their arms slightly abducted and not touching the trunk. The device applied a low-level alternating current (≤90 μA) at 5, 50, and 250 kHz to obtain impedance components, including resistance (R) and reactance (Xc), and estimate body water compartments [30,31]. Segmental values were obtained for each limb. In the present analysis, segmental lower-limb PhA at 50 kHz was used as the index of muscle quality and was calculated as PhA (°) = arctan (Xc/R) × (180/π) [24]. Lower-limb muscle mass (kg) and lower-limb PhA (degrees) were calculated as the mean of the right and left lower limbs [24].

### 2.4. TUG Test

Functional mobility was assessed using the Timed Up and Go (TUG) test, as described previously [6]. Consistent with a previously described standardized protocol [24], participants began the test in a seated position on a chair without armrests, with a seat height of 42 cm. On the start signal, participants stood up from the chair, walked 3 m at their fastest safe speed to a marker placed on the floor, turned around the marker, walked back to the chair, and sat down again. The time from the start signal to the moment when the participant sat back on the chair was recorded in seconds. All participants performed the test barefoot and without an assistive device. The test was performed twice, and the faster time was used for analysis.

### 2.5. Statistical Analysis

Statistical analyses were performed using IBM SPSS Statistics for Windows, version 29.0.1.0 (IBM Corp., Armonk, NY, USA), with statistical significance set at a two-sided *p*-value of <0.05. The distribution of each variable was assessed using the Shapiro–Wilk test. Although several variables showed non-normal distributions, no transformation was applied before the regression analyses. The multiple linear regression models were conducted using the original measured values to maintain clinical interpretability.

Multiple linear regression analyses were conducted with TUG time as the dependent variable to investigate whether lower-limb muscle mass and PhA were independently associated with functional mobility after adjusting for potential confounders. Age and sex were selected as basic demographic covariates because they may influence TUG performance, muscle mass, and PhA. Lower-limb muscle mass and lower-limb PhA were included as the main BIA-derived indices of muscle quantity and quality, respectively. BMI was included in Model 1 as a general indicator of body size, whereas height and weight were included separately in Model 2 to examine whether the associations of BIA-derived indices with TUG performance were robust to different approaches to body-size adjustment. A stepwise selection procedure was applied as an exploratory approach to identify BIA-derived variables associated with TUG performance after adjustment for potential covariates. Because stepwise procedures may yield unstable variable selection and optimistic estimates, the regression findings were interpreted as exploratory rather than confirmatory.

ROC analysis was performed to explore values for distinguishing slow TUG performance. Based on previous studies, slow TUG performance was defined using two prespecified thresholds: TUG ≥ 9 s and TUG ≥ 10.2 s. These thresholds yielded 30 cases and 250 controls for TUG ≥ 9 s and 21 cases and 259 controls for TUG ≥ 10.2 s. Areas under the curve (AUCs) with 95% confidence intervals (CIs) were reported, and exploratory values were derived using the Youden index (sensitivity + specificity − 1). In the ROC analysis, muscle quantity was indexed to body size to improve clinical interpretability and align with major sarcopenia frameworks emphasizing lean mass relative to body size (e.g., BMI-adjusted criteria in the Foundation for the National Institutes of Health Sarcopenia Project and scalable approaches endorsed by the Asian Working Group for Sarcopenia) [15,32,33], and was represented by the lower-limb muscle mass-to-body weight ratio (%), which was calculated as (mean lower-limb muscle mass/body weight) × 100. Muscle quality was represented by standardized PhA [25]. Z-scores were calculated within the present sample using age- and sex-specific strata. Additional ROC analyses were performed for age and BMI to compare their discriminatory ability with that of the BIA-derived indices. Because these ROC-derived values were derived and evaluated within the same analytical sample, without internal validation such as bootstrapping or cross-validation and without validation in an external cohort, these ROC-derived values were considered exploratory estimates. This issue was particularly relevant because the number of slow-TUG cases was limited at both thresholds.

Given that PhA varies substantially by age and sex, standardized PhA was calculated using the mean (μ) and standard deviation (σ) within each age-by-sex stratum as follows: Z = (PhA − μ_age, sex)/σ_age, sex. Age-specific strata were defined a priori, based on published age-related PhA patterns. A large systematic review and meta-analysis have shown that PhA stabilizes in adulthood (approximately 18–38 years in men and 18–48 years in women) and decreases progressively with advancing age [26]. A cohort study involving adults aged ≥ 50 years reported that the mean PhA was lower in adults aged > 65 years than in those aged 50–65 years [34]. Accordingly, the following age groups were defined for Z-score standardization: stable period (20 to <47 years), declining period 1 (48 to <64 years), and declining period 2 (≥65 years). Z-score values identified in the ROC analysis were back-transformed to absolute PhA values (degrees) for each age-by-sex stratum using the following equation: back-transformed PhA value = μ_age, sex + (Z_ROC-derived value × σ_age, sex). Because one age-by-sex stratum included a very small number of participants, analyses using standardized PhA and back-transformed PhA values were also interpreted as exploratory.

## 3. Results

### 3.1. Participants and Baseline Characteristics

The baseline anthropometric and functional characteristics are summarized in Table 1. The participants had a mean age of 59.60 ± 17.50 years (median, 65.00 years). In the total sample, the mean lower-limb PhA and muscle mass-to-body weight ratio were 4.50 ± 0.80° and 12.10 ± 1.70%, respectively, and the median TUG time was 6.20 s (interquartile range: 5.20–7.50 s).

### 3.2. Association Between TUG Performance and Body Composition Variables

The results of the multiple regression analyses for the association of TUG time with age, body size, lower-limb muscle mass, and lower-limb PhA are presented in Table 2. In Model 1, longer TUG time was associated with older participants (unstandardized regression coefficient [B] = 0.03, standardized regression coefficient [β] = 0.24, *p* < 0.001) and higher BMI (B = 0.14, β = 0.24, *p* < 0.001), whereas shorter TUG time was associated with higher lower-limb PhA (B = −0.70, β = −0.28, *p* < 0.001) and greater lower-limb muscle mass (B = −0.24, β = −0.17, *p* = 0.009). In Model 2, where BMI was replaced by height and weight, TUG time was significantly associated with age (B = 0.02, β = 0.20, *p* = 0.002), height (B = −0.09, β = −0.33, *p* < 0.001), weight (B = 0.05, β = 0.23, *p* < 0.001), and lower-limb PhA (B = −0.79, β = −0.31, *p* < 0.001). Sex was entered as a candidate variable in both regression models but was not retained in the final models because it did not meet the stepwise selection criterion. Specifically, sex was not significantly associated with TUG performance after accounting for the retained variables in Model 1 (*p* = 0.996) or Model 2 (*p* = 0.713). The non-retention of lower-limb muscle mass in Model 2 may reflect differences in body-size adjustment, because height and weight were entered separately and may have captured variance shared with absolute lower-limb muscle mass.

### 3.3. ROC Analysis and Exploratory Values for Slow TUG Performance

**Figure 1:**
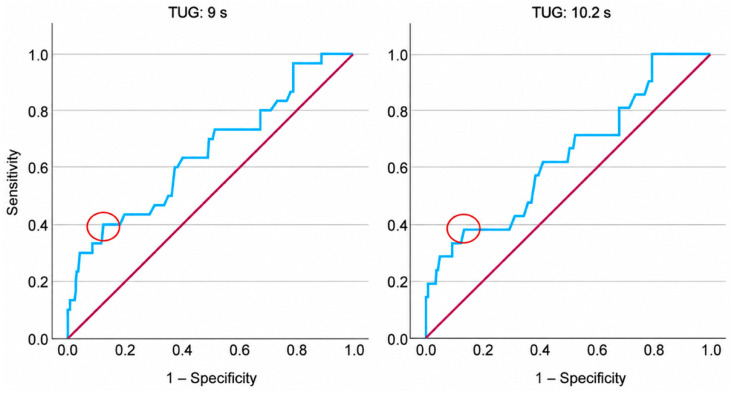
Receiver operating characteristic curves for the lower-limb muscle mass-to-body weight ratio for discriminating slow Timed Up and Go performance. The left and right panels show the receiver operating characteristic curves for TUG ≥ 9 s and TUG ≥ 10.2 s, respectively. The solid light-blue line indicates the receiver operating characteristic curve, and the purple diagonal line indicates no-discrimination performance (AUC = 0.50). The red circle indicates the exploratory value derived using the Youden index. For TUG ≥ 9 s, the AUC was 0.75 and the Youden index-derived value was 11.295% (sensitivity, 0.73; specificity, 0.71). For TUG ≥ 10.2 s, the AUC was 0.78 and the Youden index-derived value was 10.962% (sensitivity, 0.76; specificity, 0.76). The *y*-axis and *x*-axis represent sensitivity and 1 − specificity, respectively. Abbreviations: AUC, area under the curve; TUG, Timed Up and Go.

**Figure 2:**
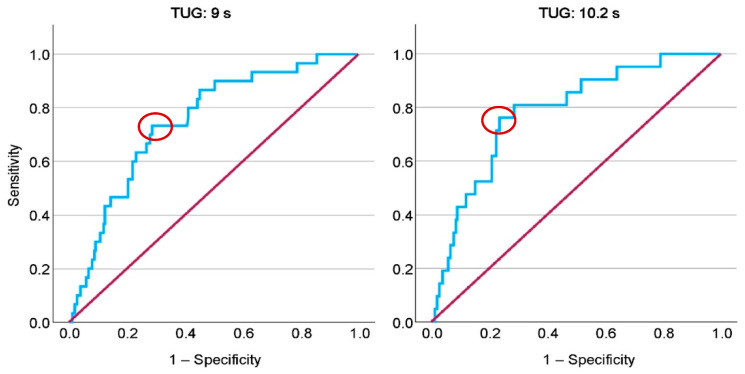
Receiver operating characteristic curves for standardized lower-limb phase angle for discriminating slow Timed Up and Go performance. Standardized lower-limb phase angle indicates the age- and sex-specific Z-score for lower-limb phase angle. The left and right panels show the receiver operating characteristic curves for TUG ≥ 9 s and TUG ≥ 10.2 s, respectively. The solid light-blue line indicates the receiver operating characteristic curve, and the purple diagonal line indicates no-discrimination performance (AUC = 0.50). The red circle indicates the exploratory value derived using the Youden index. For TUG ≥ 9 s, the AUC was 0.65 and the Youden index-derived value was Z = −1.04 (sensitivity, 0.40; specificity, 0.88). For TUG ≥ 10.2 s, the AUC was 0.64 and the Youden index-derived value was Z = −1.04 (sensitivity, 0.38; specificity, 0.87). The *y*-axis and *x*-axis represent sensitivity and 1 − specificity, respectively. Abbreviations: AUC, area under the curve; PhA, phase angle; TUG, Timed Up and Go.

Because the number of participants classified as having slow TUG performance was limited, particularly for the TUG ≥ 10.2 s threshold, the ROC-derived values should be interpreted cautiously as exploratory estimates rather than definitive clinical thresholds. The results of the ROC analysis and exploratory values derived using the Youden index are presented in Table 3 and Figure 1 and Figure 2. For TUG ≥ 9 s, the AUC for the lower-limb muscle mass-to-body weight ratio was 0.75 (95% CI: 0.66–0.84), with a Youden index-derived value of 11.295% (sensitivity: 0.73, specificity: 0.71). The corresponding AUC for standardized lower-limb PhA was 0.65 (95% CI: 0.54–0.76), with a Youden index-derived value of Z = −1.04 (sensitivity: 0.40, specificity: 0.88).

For TUG ≥ 10.2 s, the AUC for the lower-limb muscle mass-to-body weight ratio was 0.78 (95% CI: 0.69–0.88), with a Youden index-derived value of 10.962% (sensitivity: 0.76, specificity: 0.76). The corresponding AUC for standardized lower-limb PhA was 0.64 (95% CI: 0.51–0.77), with a Youden index-derived value of Z = −1.04 (sensitivity: 0.38, specificity: 0.87).

Additional ROC analyses showed that age had moderate discriminatory ability for slow TUG performance, with AUCs of 0.74 for TUG ≥ 9 s and 0.76 for TUG ≥ 10.2 s. BMI showed lower discriminatory ability, with AUCs of 0.63 and 0.65, respectively. These comparisons were also exploratory and were performed only to contextualize the discriminatory ability of the BIA-derived indices.

### 3.4. Back-Transformed Lower-Limb PhA Values for Interpretability

The standardized lower-limb PhA value identified in the ROC analysis was back-transformed into absolute PhA values only to aid interpretation of the standardized estimate. These back-transformed values are provided in Supplementary Table S1. Because they were calculated from age- and sex-specific strata within the same sample, including strata with small numbers of participants, they should not be interpreted as validated age- and sex-specific screening thresholds or reference values.

## 4. Discussion

This study examined the associations of lower-limb muscle mass and PhA with TUG performance in community-dwelling Japanese adults. The findings revealed that lower-limb PhA and lower-limb muscle mass were independently associated with faster TUG performance after adjusting for age and body size. In the ROC analysis, the lower-limb muscle mass-to-body weight ratio showed moderate discriminatory ability for slow TUG performance (AUC, 0.75–0.78), whereas standardized lower-limb PhA showed only modest discriminatory ability (AUC, 0.64–0.65). Additional ROC analyses showed that age had moderate discriminatory ability for slow TUG performance (AUC, 0.74–0.76), whereas BMI showed lower discriminatory ability (AUC, 0.63–0.65). These findings indicate that age is an important discriminator of slow TUG performance, as expected, and that BIA-derived indices may provide muscle-related information that complements demographic factors. However, the ROC-derived values should be interpreted as exploratory estimates rather than definitive clinical thresholds.

A plausible explanation for these findings is that absolute lower-limb muscle mass is strongly influenced by body size. In Model 1, BMI was used as a general indicator of body size, and lower-limb muscle mass remained independently associated with TUG performance. However, in Model 2, height and weight were entered separately instead of BMI. As height and weight are directly related to absolute muscle mass, these variables may have captured part of the variance shared between body size and lower-limb muscle mass. Consequently, the independent contribution of absolute lower-limb muscle mass was attenuated and not retained in the final model. Importantly, multicollinearity was not substantial, as all VIF values were <5, suggesting that this finding was more likely due to differences in body-size modeling than to severe collinearity.

In contrast, lower-limb PhA remained independently associated with TUG performance in both models. PhA is calculated from resistance and reactance and is considered to reflect qualitative tissue properties, including cellular integrity, cell membrane function, and fluid distribution [13]. Therefore, PhA may provide information on muscle quality that is not fully captured by muscle mass or body size. This interpretation is consistent with previous reports indicating that functional decline may be disproportionate to muscle mass loss and that qualitative changes in skeletal muscle, such as adipose tissue infiltration and reduced muscle quality, contribute to mobility limitations [17,18,35]. In addition, previous studies have reported that lower-limb PhA is independently associated with mobility outcomes, including TUG performance, and may provide information that complements muscle mass measures [24,30]. Taken together, the present findings suggest that segmental lower-limb PhA may be associated with mobility-related muscle quality even when body size is accounted for using different modeling approaches.

Regarding muscle quality, age- and sex-standardized lower-limb PhA showed only modest discriminatory ability, with relatively high specificity but low sensitivity at both TUG thresholds. Therefore, markedly low standardized PhA may help characterize individuals with poorer mobility-related muscle quality within the same demographic stratum, but it is not suitable as a stand-alone screening marker for slow TUG performance. Importantly, statistical association and ROC discrimination do not directly imply clinical utility. Although lower-limb PhA was independently associated with TUG performance, the modest AUC values, low sensitivity, cross-sectional design, and lack of validation indicate that the ROC-derived values should be regarded strictly as exploratory, hypothesis-generating estimates. These values should not be used to classify individuals, guide clinical decision-making, or support community screening at this stage. Further validation in larger independent cohorts is required before any clinical interpretation or application of these values can be considered.

A meta-analysis evaluating PhA for sarcopenia screening reported a recommended cutoff interval of approximately 4.54–5.25° and identified ethnicity and BMI as key sources of heterogeneity [21]. These observations support the biological plausibility that demographic and body-size-related factors influence PhA; accordingly, we standardized PhA by age and sex and explicitly examined body-size modeling in our analyses. Previous studies have primarily employed whole-body PhA as an index, whereas the present study used lower-limb PhA. As lower-limb PhA tends to be lower than whole-body PhA [30], the back-transformed lower-limb PhA values in the present study were generally lower than values reported in previous studies that used whole-body PhA [21]. The partial overlap between our back-transformed lower-limb PhA values and the previously reported interval may reflect differences in segmental versus whole-body assessment, population characteristics, body size, and device-specific algorithms [21]. However, these back-transformed values were provided only for interpretability and should not be regarded as validated reference values or clinical thresholds.

The findings may have practical implications, but they should be interpreted cautiously. TUG is a simple, low-cost functional test and should remain a practical tool for assessing mobility performance. The present findings do not suggest that BIA should replace TUG. Although lower-limb PhA and muscle mass were associated with TUG performance, TUG is a multidimensional mobility task that requires muscle quantity and quality as well as balance, lower-limb coordination, turning ability, agility, and other neuromotor functions. Therefore, BIA-derived indices should not be interpreted as comprehensive determinants or substitutes for TUG performance. Rather, lower-limb muscle mass and PhA may provide complementary information on muscle-related aspects of mobility performance. Future studies should determine whether BIA-derived lower-limb indices improve risk stratification beyond conventional demographic and performance-based assessments and whether intervention-induced changes in segmental lower-limb PhA translate into clinically meaningful improvements in mobility outcomes.

This study has several limitations. First, its cross-sectional design precludes causal or predictive inferences. Although lower-limb PhA and lower-limb muscle mass were associated with TUG performance, the present findings do not indicate whether low PhA or low muscle mass causes slower TUG performance or predicts future mobility decline.

Second, the participants were independently ambulatory volunteers recruited using convenience sampling from limited regions in Niigata Prefecture, Japan. The sample was also highly imbalanced by sex, and some age-by-sex strata were small, particularly men aged 48–64 years. These factors may reduce the stability of the standardized PhA estimates and back-transformed PhA values and limit generalizability to frailer individuals, clinical populations, non-Japanese populations, or more balanced cohorts. Because of this sample imbalance, sex- or age-by-sex-stratified regression analyses were not performed, as such analyses would have produced unstable estimates and may have increased the risk of misleading interpretation.

Third, the regression and ROC analyses were exploratory. Stepwise variable selection was used only as an exploratory variable-selection procedure and may yield unstable selected models, overfitted estimates, and optimistic apparent associations, particularly when candidate predictors are correlated or when body-size and BIA-derived variables share variance. Therefore, the regression findings should be interpreted as exploratory associations rather than confirmatory evidence. In addition, the ROC-derived values were derived and evaluated in the same sample without internal validation, such as bootstrapping or cross-validation, or external validation in an independent cohort. Moreover, the number of slow-TUG cases was limited, with only 30 cases for TUG ≥ 9 s and 21 cases for TUG ≥ 10.2 s. This limited number of cases may reduce the precision, stability, and reproducibility of the exploratory ROC-derived values, particularly those based on the Youden index. Therefore, these ROC-derived values should be considered hypothesis-generating estimates and should not be used as clinical screening thresholds until validated in larger independent cohorts.

Fourth, BIA-derived PhA and muscle mass may be influenced by hydration status, recent food or fluid intake, recent physical activity, skin temperature, electrode contact, and device-specific estimation algorithms. In addition, BIA-derived muscle mass was not validated against reference methods, such as dual-energy X-ray absorptiometry or magnetic resonance imaging, within the present study. Although all assessments were conducted by trained staff under temperature-controlled indoor conditions using the same multifrequency segmental BIA device and standardized measurement procedures, the muscle mass values should be interpreted as device-specific BIA-derived estimates rather than direct measurements of muscle mass.

Fifth, residual confounding is possible because several factors that may influence both BIA-derived indices and TUG performance, such as physical activity, nutritional status, comorbidities, medication use, and other lifestyle-related variables, were not fully assessed. TUG performance is also influenced by multiple functional components, including balance, lower-limb coordination, turning ability, agility, and neuromotor control, which were not directly assessed. Therefore, the observed associations should be interpreted as muscle-related correlates of mobility performance rather than evidence that BIA-derived indices fully explain or predict TUG performance.

Finally, TUG was used as the sole performance-based mobility outcome. Therefore, the present findings may not extend to other mobility measures, such as gait speed, chair stand performance, balance tests, or composite physical performance measures. Future studies should examine multiple mobility outcomes and validate or refine these exploratory ROC-derived values using larger independent cohorts, internal validation procedures, and population-specific clinically relevant criteria.

## 5. Conclusions

This study showed that lower-limb BIA-derived indices were associated with TUG performance in community-dwelling Japanese adults. Segmental lower-limb PhA was independently associated with TUG performance across alternative body-size adjustment models, suggesting that it may reflect mobility-related aspects of muscle quality beyond body size and muscle quantity. The lower-limb muscle mass-to-body weight ratio showed moderate discriminatory ability for slow TUG performance, whereas standardized lower-limb PhA showed only modest discrimination with low sensitivity. The ROC-derived values should be interpreted strictly as exploratory, hypothesis-generating estimates because they were derived and evaluated in the same sample. External validation across independent cohorts and devices is required before these values can be considered for community screening or clinical decision-making.

## Figures and Tables

**Table 1:** Participant characteristics and results of normality testing.

Variable	*n*	Mean ± SD or Count	Median (IQR)	Shapiro–Wilk *p*	Normality
Sex (male/female)	280 (men, 43/women, 237)	–	–	–	–
Age (years)	280	59.60 ± 17.50	65.00 (48.00–72.20)	<0.001	No
Height (cm)	280	157.70 ± 7.80	157.00 (152.00–162.10)	<0.001	No
Weight (kg)	280	57.10 ± 10.50	55.00 (49.60–63.60)	<0.001	No
BMI (kg/m^2^)	280	22.90 ± 3.60	22.30 (20.30–25.10)	<0.001	No
Lower-limb muscle mass (kg)	280	6.90 ± 1.50	6.60 (5.90–7.40)	<0.001	No
Lower-limb PhA (°)	280	4.50 ± 0.80	4.50 (4.00–4.90)	<0.001	No
Lower-limb muscle mass-to-body weight ratio (%)	280	12.10 ± 1.70	12.10 (10.80–13.20)	0.166	Yes
TUG (s)	280	6.60 ± 2.10	6.20 (5.20–7.50)	<0.001	No

Values are presented as mean ± standard deviation (SD) or as median (interquartile range [IQR]), as appropriate; sex is presented as counts. Normality was assessed using the Shapiro–Wilk test. Abbreviations: BMI, body mass index; PhA, phase angle; TUG, Timed Up and Go.

**Table 2:** Multiple regression models of factors associated with TUG performance.

Variable	Model 1			Model 2		
	Model 1 B	β	*p* Value	Model 2 B	β	*p* Value
Sex	–	–	–	–	–	–
Age (years)	0.03	0.24	<0.001	0.02	0.20	0.002
BMI (kg/m^2^)	0.14	0.24	<0.001	–	–	–
Height (cm)	–	–	–	−0.09	−0.33	<0.001
Weight (kg)	–	–	–	0.05	0.23	<0.001
Lower-limb PhA (°)	−0.70	−0.28	<0.001	−0.79	−0.31	<0.001
Lower-limb muscle mass (kg)	−0.24	−0.17	0.009	–	–	–
Constant	6.66	–	<0.001	19.81	–	<0.001

TUG time (s) was the dependent variable. Model 1 included age, sex, BMI, lower-limb muscle mass, and lower-limb PhA as candidate predictors. Model 2 replaced BMI with height and weight and included age, sex, height, weight, lower-limb muscle mass, and lower-limb PhA as candidate predictors. B denotes the unstandardized regression coefficient, whereas β denotes the standardized regression coefficient. Model fit statistics are as follows: Model 1 (*n* = 280), *R*^2^ = 0.364, adjusted *R*^2^ = 0.355, SEE = **1.661**, *F*(4, 275) = 39.37, *p* < 0.001, Durbin–Watson = 1.61. Model 2 (*n* = 280), *R*^2^ = 0.376, adjusted *R*^2^ = 0.367, SEE = **1.645**, *F*(4, 275) = 41.46, *p* < 0.001, Durbin–Watson = 1.60. Collinearity in the final models was acceptable (all VIFs < 5; maximum VIF = **2.20** for Model 1 and **1.84** for Model 2). Sex was entered as a candidate variable but was not retained in the final models (Model 1: *p* = 0.996; Model 2: *p* = 0.713). Abbreviations: BMI, body mass index; PhA, phase angle; SEE, standard error of the estimate; VIF, variance inflation factor; TUG, Timed Up and Go.

**Table 3:** ROC analysis and exploratory values of BIA-derived indices, age, and BMI for slow TUG performance.

TUG Threshold	Slower than TUG Standards (%)	Predictor	AUC	95% CI	Exploratory Value	Sensitivity	Specificity	Youden Index
9 s	(30/280 = 10.79%)	Muscle mass-to-body weight ratio (%)	0.75	0.66–0.84	11.295	0.73	0.71	0.45
		Standardized PhA (*Z*-score)	0.65	0.54–0.76	−1.04	0.40	0.88	0.28
		Age (years)	0.74	0.65–0.82	65.5	0.80	0.54	0.34
		BMI (kg/m^2^)	0.63	0.52–0.73	22.496	0.70	0.55	0.25
10.2 s	(21/280 = 7.55%)	Muscle mass-to-body weight ratio (%)	0.78	0.69–0.88	10.962	0.76	0.76	0.53
		Standardized PhA (*Z*-score)	0.64	0.51–0.77	−1.04	0.38	0.87	0.25
		Age (years)	0.76	0.66–0.86	69.5	0.71	0.67	0.38
		BMI (kg/m^2^)	0.65	0.53–0.78	23.673	0.67	0.65	0.32

Slow TUG performance was defined using two prespecified thresholds: TUG ≥ 9 s and TUG ≥ 10.2 s. Exploratory values were derived using the Youden index and should not be interpreted as validated clinical thresholds. Standardized PhA indicates the age- and sex-specific Z-score for lower-limb PhA. Additional ROC analyses were performed for age and BMI to contextualize their discriminatory ability relative to that of the BIA-derived indices. Abbreviations: AUC, area under the receiver operating characteristic curve; BMI, body mass index; CI, confidence interval; PhA, phase angle; ROC, receiver operating characteristic; TUG, Timed Up and Go.

## Data Availability

The dataset is available from the authors upon reasonable request.

## Supplementary Materials

The following supporting information can be downloaded at: https://www.mdpi.com/article/10.3390/jcm15114388/s1. Table S1: Back-transformed lower-limb PhA values for interpretability.

## Abbreviations

AUC	area under the curve
B	unstandardized regression coefficient
BIA	bioelectrical impedance analysis
BMI	body mass index
CI	confidence interval
PhA	phase angle
ROC	receiver operating characteristic
TUG	Timed Up and Go
VIF	variance inflation factor
Z	standardized score
β	standardized regression coefficient
