# Supplementary material for: Lower-Limb Phase Angle and Muscle Mass Ratio Are Associated with Slow Timed Up and Go Performance in Community-Dwelling Japanese Adults
Source: J Clin Med. 2026 Jun 5;15(11):4388. doi: 10.3390/jcm15114388 (PMC13258569; doi:10.3390/jcm15114388)
Supplement: Supplementary file 1 [file jcm-15-04388-s001.zip › jcm-4313656-supplementary.pdf]

Supplementary Table S1. Back-transformed lower-limb PhA values for interpretability.

Age group	Sex	*n*	Mean PhA (°)	SD PhA (°)	Back-transformed PhA for TUG ≥9 s (°)	Back-transformed PhA for TUG ≥10.2 s (°)
20–47	Men	17	6.15	0.73	5.39	5.39
20–47	Women	52	4.82	0.58	4.21	4.21
48–64	Men	4	5.80	0.58	5.20	5.20
48–64	Women	66	4.62	0.53	4.07	4.07
≥65	Men	22	4.73	0.83	3.87	3.87
≥65	Women	119	4.03	0.59	3.41	3.41

Note: Back-transformed lower-limb PhA values were calculated only to aid interpretation of the standardized PhA estimate identified in the ROC analysis. These values were derived from age- and sex-specific strata within the present sample and should not be interpreted as validated age- and sex-specific screening thresholds, clinical cutoff values, or reference values.

Abbreviations: PhA, phase angle; ROC, receiver operating characteristic; SD, standard deviation; TUG, Timed Up and Go.
